# Novel Genetic Dysregulations and Oxidative Damage in *Fusarium graminearum* Induced by Plant Defense Eliciting Psychrophilic *Bacillus atrophaeus* TS1

**DOI:** 10.3390/ijms222212094

**Published:** 2021-11-09

**Authors:** Muhammad Zubair, Ayaz Farzand, Faiza Mumtaz, Abdur Rashid Khan, Taha Majid Mahmood Sheikh, Muhammad Salman Haider, Chenjie Yu, Yujie Wang, Muhammad Ayaz, Qin Gu, Xuewen Gao, Huijun Wu

**Affiliations:** 1 Department of Plant Pathology, College of Plant Protection, Nanjing Agricultural University, Key Laboratory of Integrated Management of Crop Diseases and Pests, Ministry of Education, Nanjing 210095, China; zubair_biotech@yahoo.com (M.Z.); ayaz.farzand@uaf.edu.pk (A.F.); malix.477@gmail.com (A.R.K.); tahamajid1705@yahoo.com (T.M.M.S.); yuchenjie0501@163.com (C.Y.); 2019202005@njau.edu.cn (Y.W.); m.ayazbiotech@gmail.com (M.A.); guqin@njau.edu.cn (Q.G.); gaoxw@njau.edu.cn (X.G.); 2 Department of Plant Pathology, University of Agriculture, Faisalabad 38040, Pakistan; 3 Department of Pharmacology, School of Medicine, Tehran University of Medical Sciences, P.O. Box 13145-784, Tehran 13145-784, Iran; mumtaz.faiza@yahoo.com; 4 College of Horticulture, Nanjing Agricultural University, Nanjing 210095, China; salman.hort1@gmail.com

**Keywords:** psychrophilic, biotic/abiotic stresses, biochemical, genetic dysregulations, necrosis inducing proteins, plant defense induction

## Abstract

This study elaborates inter-kingdom signaling mechanisms, presenting a sustainable and eco-friendly approach to combat biotic as well as abiotic stress in wheat. *Fusarium graminearum* is a devastating pathogen causing head and seedling blight in wheat, leading to huge yield and economic losses. Psychrophilic *Bacillus atrophaeus* strain TS1 was found as a potential biocontrol agent for suppression of *F. graminearum* under low temperature by carrying out extensive biochemical and molecular studies in comparison with a temperate biocontrol model strain *Bacillus amyloliquefaciens* FZB42 at 15 and 25 °C. TS1 was able to produce hydrolytic extracellular enzymes as well as antimicrobial lipopeptides, i.e., surfactin, bacillomycin, and fengycin, efficiently at low temperatures. The *Bacillus* strain-induced oxidative cellular damage, ultrastructural deformities, and novel genetic dysregulations in the fungal pathogen as the bacterial treatment at low temperature were able to downregulate the expression of newly predicted novel fungal genes potentially belonging to necrosis inducing protein families (*fgHCE* and *fgNPP1*). The wheat pot experiments conducted at 15 and 25 °C revealed the potential of TS1 to elicit sudden induction of plant defense, namely, H_2_O_2_ and callose enhanced activity of plant defense-related enzymes and induced over-expression of defense-related genes which accumulatively lead to the suppression of *F. graminearum* and decreased diseased leaf area.

## 1. Introduction

Biotic and abiotic stresses contribute towards the reduction of growth and yield in cereal crops. The phytopathogenic fungus *Fusarium graminearum* has deleterious effects on major cereal crops including wheat. The fungus is responsible for growth and yield losses in wheat by instigating *Fusarium* head blight (FHB) and seedling blight disease [1]. In addition to yield loss, the fungus produces a vast array of mycotoxins, i.e., zearalenone (ZEN) and deoxynivalenol (DON), which can have detrimental health effects on the organisms consuming them [2]. Despite significant economic and health losses, there has been a lack of FHB resistant wheat cultivars, and toxic fungicides are the only viable option to control the fungus, against which it has started to develop resistance [3,4]. Alternatively, the use of biocontrol rhizospheric bacteria or their products has emerged as an effective, sustainable, and eco-friendly approach for the control of phytopathogens [5].

Among rhizospheric bacteria, *Bacillus* spp. are reported to be the most efficient biocontrol agents. These bacteria have the ability to survive and colonize complex environments and to produce a wide range of antifungal extracellular enzymes as well as antimicrobial secondary metabolites including lipopeptides (LPs) [6,7]. *Bacillus* can produce multiple low molecular-weight, amphiphilic, antimicrobial compounds synthesized by non-ribosomal peptide synthetase (NRPS) enzyme-complexes such as surfactin, iturins, and fengycin [8,9]. These lipopeptides can efficiently halt the growth of phytopathogens; a previous study emphasized that fengycin produced by *Bacillus* spp. is able to suppress *F. graminearum* [10] associated with the control of FHB in wheat via direct antagonism. This fengycin also elicited induced systemic resistance (ISR) in tomato plants against *Sclerotinia sclerotiorum* [11]. Similarly, bacillomycin D from *Bacillus* sp. has been reported to induce the suppression of *F. graminearum* in wheat [12].

The LPs produced by *Bacillus subtilis* can cause fungal cell death through the induction of oxidative stress [11,13]. Moreover, the treatment of *Bacillus* spp. can lead to structural and functional deterioration in *F. graminearum* as studied through electron microscopy and expression regulation of fungal pathogenicity-linked genes [14,15,16]. A fungal genome-wide analysis can be performed to identify pathogenicity-associated gene families and their phylogenetic relationship, gene structure, and biochemical properties [17]. This study presents the first report of such an analysis and prediction of eight new genes belonging to necrosis-inducing protein families *HCE* and *NPP1* of *F. graminearum*. The expression analysis of newly predicted and already reported pathogenicity-linked fungal genes in response to *Bacillus* treatment was also studied in the present work.

In addition to direct antagonism towards the pathogen through the production of several secondary metabolites [18,19,20], the LPs also serve as the latest class of pathogen-associated Molecular patterns (PAMPs), eliciting plant immune response. The plant defense is initiated by the colonizing *Bacillus* spp. by the means of ISR; the LPs synthesized by rhizospheric bacteria can function as elicitors for the stimulation of plant defense responses [21,22]. The priming of plants with biocontrol bacteria can trigger an orchestrated activation of several plant defense responses including oxidative defense [23], secondary metabolites production [24], enhanced activity of defense-related enzymes, and expression upregulation of defense-related genes in plants [25].

Several studies have focused on the biocontrol of plant diseases, but the present study explores the mechanism of action of psychrophilic *Bacillus atrophaeus* strain TS1 to suppress *F. graminearum* in plants at optimal growth conditions as well as at cold temperature. The strain TS1, formerly isolated from Qinghai-Tibaten Plateau, was selected as the biocontrol strain. This strain was identified through phenotypic characters and genome sequencing through 16s rRNA [26] and was evaluated for secondary metabolites production, extracellular hydrolytic enzymes, and lipopeptides under low temperatures. In addition, the strain was also evaluated to check how these products enable bacteria to induce structural, functional, and novel genetic dysregulations in the pathogenic fungus *F. graminearum*. The current study offers unique insights into the use of the psychrophilic *Bacillus* strain TS1 as an elicitor of the defense response, resulting in plant disease control at low temperature and enabling the plants to combat biotic as well as abiotic stress simultaneously.

## 2. Results

### 2.1. Fungal Inhibition at Low Temperature

**Figure 1:**
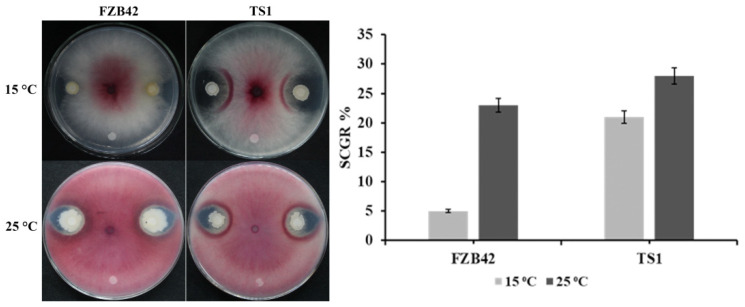
Antifungal activity of *Bacillus* strains FZB42 and TS1 against *F. graminearum* on potato dextrose agar (PDA) medium. The pink (fungus growth) was grown at normal temperature 25°C and the whitish color was grown at cold temperature 15 °C after being treated with both strains. The strain TS1 shows clear inhibition zones at cold temperature as compared to strain FZB42. The graph represents the conidial suppression by *Bacillus* strains at 15 and 25 °C shown as SCGR %. The error bars represent the standard error of the mean (n = 5). The significant difference among the treatments was observed by using Turkey’s HSD test at *p* ≤ 0.05.

The result of fungal inhibition showed that the mesophilic *Bacillus amyloliquefaciens* strain FZB42 and psychrophilic *Bacillus atrophaeus* strain TS1 were able to significantly inhibit the growth of *F. graminearum* at 25 °C after 4 days. The diameter for the clear zone of inhibition for FZB42 and TS1 against the fungal pathogen was observed to be 9 mm and 14 mm, respectively. Whereas, under cold temperature, i.e., 15 °C, TS1 showed very good inhibition of around 14 mm and FZB42 was not able to significantly suppress fungal growth (Figure 1). Similar results were observed for the suppression of conidial germination rate (SCGR %). At 25 °C, both strains, FZB42 and TS1, were able to significantly suppress the conidial germination by 23% and 28%, respectively (Figure 1). Whereas, in contrast to FZB42, the psychrophilic strain TS1 also significantly suppressed fungal conidia by 21% under cold temperature.

### 2.2. Biocontrol Determinants under Cold Stress

**Figure 2:**
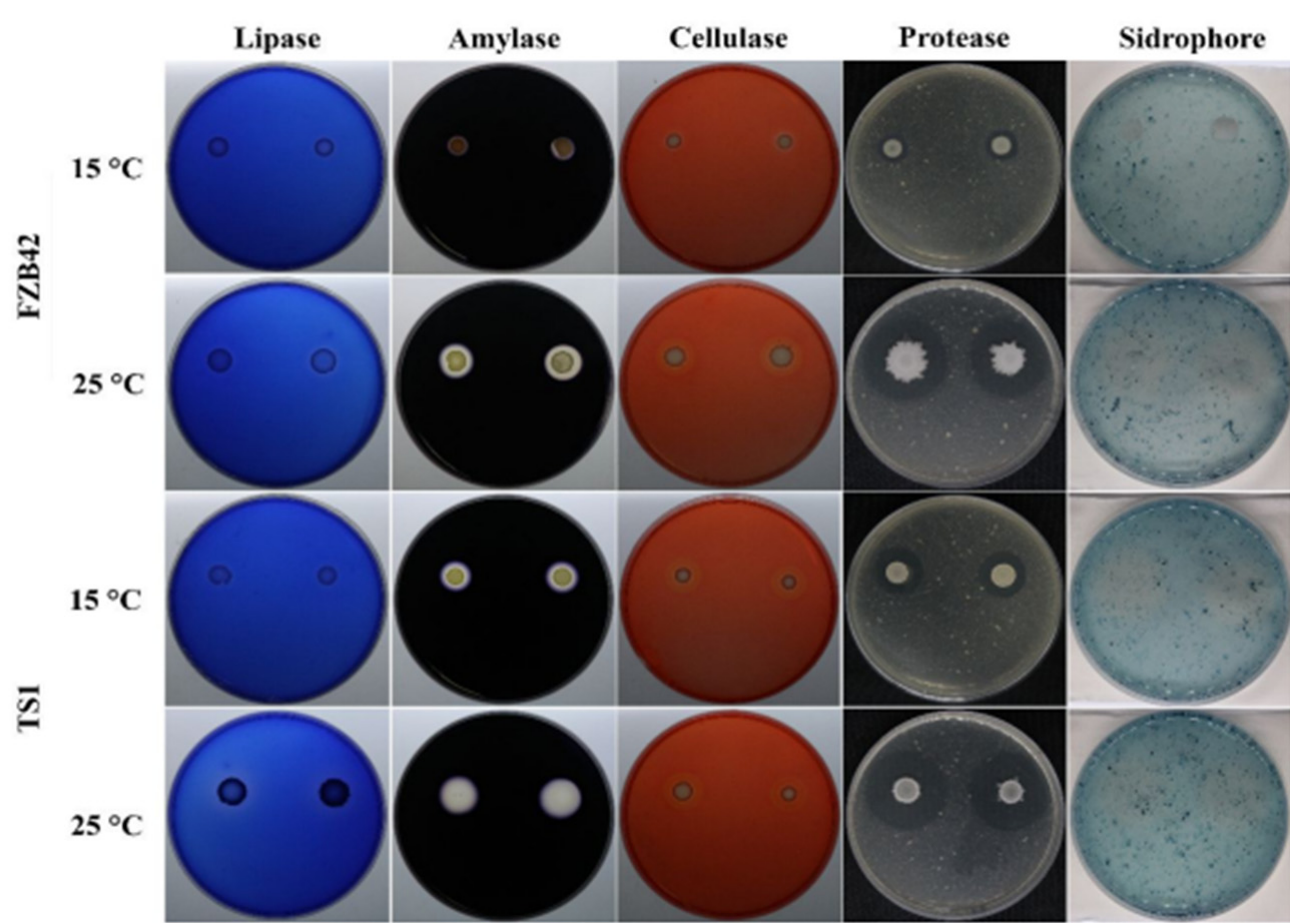
In vitro screening of *Bacillus* strains FZB42 and TS1 for biochemical (lipase, amylase, cellulase, protease, and siderophores) production of biocontrol determinants at normal 25 °C and cold temperature 15 °C.

The screening for hydrolytic/extracellular enzymes in both *Bacillus* strains FZB42 and TS1 showed varied production ability. Both strains showed significant production of extracellular enzymes, i.e., lipase, amylase, cellulase, and protease at 25 °C as indicated by the halo zone formation around the bacterial culture on their respective media (Figure 2). The yellowish discoloration zone around the bacterial cultures indicated the production of iron-chelating compound siderophores in both strains at the optimum growth temperature. The *Bacillus* strain TS1 showed significantly higher production of all these biocontrol determinants under colder conditions as compared to FZB42, whose ability to produce these compounds at 15 °C was halted.

### 2.3. LC-MS Analysis and Expression Profiling of Lipopeptides Production under Cold Stress

The LC-MS analysis detected the production of multiple homologues of important antimicrobial lipopeptides (LPs) in *Bacillus* strains FZB42 and TS1. Both strains were able to produce a major class of LPs, i.e., bacillomycin, surfactin, and fengycin at 25 °C. Whereas, the intensity of production for FZB42 dropped significantly at 15 °C as shown by the peak intensity in the chromatograms. TS1 was able to produce three homologues of bacillomycin with *m*/*z* 1057.56, 1071.58, and 1085.59. It produced two homologues of Surfactin with *m*/*z* 1008.65 and 1022.67. This strain was also able to produce significant amounts of five homologues of fengycin for which the *m*/*z* ratio was between 1435.76 and 1505.83. For the TS1 strain, all of these compounds had reasonably high peak intensity at regular as well as cold temperatures. The LC-MS results also showed very minute differences in the retention times of the chromatogram peaks for these compounds in the TS1 strain at regular as well as at low temperatures.

The *Bacillus* strains produced high amounts of all three LPs at regular growth temperature, i.e., 25 °C, as indicated by the intensity of the peaks. The LC-MS analysis of FZB42 showed detection of five homologues of Bacillomycin with *m*/*z* 1031.54, 1045.55, 1057.56, 1059.57, and 1073.58 with high peak intensity. In addition to two homologues of surfactin produced by TS1, the strain FZB42 also produced considerably high amounts of surfactin homologues with *m*/*z* 994.64, 1036.67, and 1050.69. Similarly, at 25 °C, FZB42 produced all homologues of fengycin as produced by TS1. Whereas, at 15 °C, the LC-MS analysis of FZB42 showed significantly lower amounts of the produced LPs with much lower peak intensities as compared to the high production at regular temperature (Figure S1 and Table S1). Interestingly, the analysis detected the absence of bacillomycin homologues with *m*/*z* 1057.56 and fengycin homologues with *m*/*z* 1449.78 in the FZB42 sample grown at 15 °C. The results also suggested an increased concentration of LPs in FZB42 at 25 °C compared to 15 °C, especially fengycin, which had a much-increased peak area in the chromatogram of the sample at 25 °C as compared to the sample grown at 15 °C.

**Figure 3:**
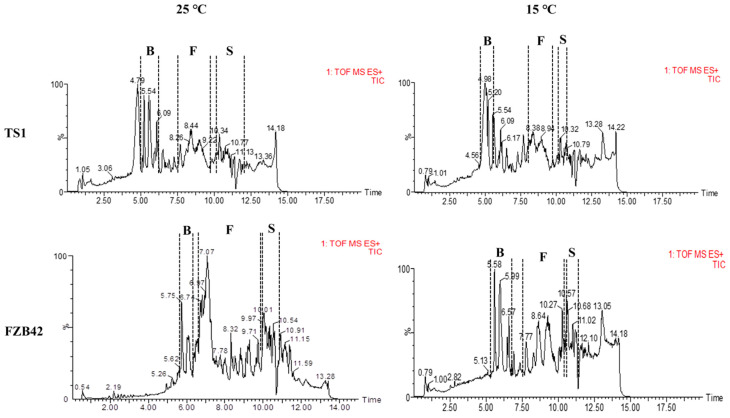
The LC-MS chromatograms show the production of LPs at different temperatures (25 and 15 °C) in *Bacillus* strains FZB42 and TS1. The dotted lines indicate the retention times of B = bacillomycin, F = fengycin, and S = surfactin in both strains.

Similarly, the expression analysis of genes encoding LPs, i.e., bacillomycin, surfactin, and fengycin, carried out at regular and cold temperatures showed that *Bacillus* strain TS1 did not have any significant downregulation in the expression of fengycin and bacillomycin at 15 °C, whereas the expression of surfactin at 15 °C was a bit lower (Figure 3). The transcriptional regulation of LPs in FZB42 at 15 °C showed significant downregulation in the expression of bacillomycin, surfactin, and fengycin encoding genes. The most downregulated gene was fengycin, which had significantly lower expression in FZB42 when grown at 15 °C as compared to the control, i.e., at regular temperature.

### 2.4. Bacillus Induced ROS Production in F. graminearum under Low Temperature

**Figure 4:**
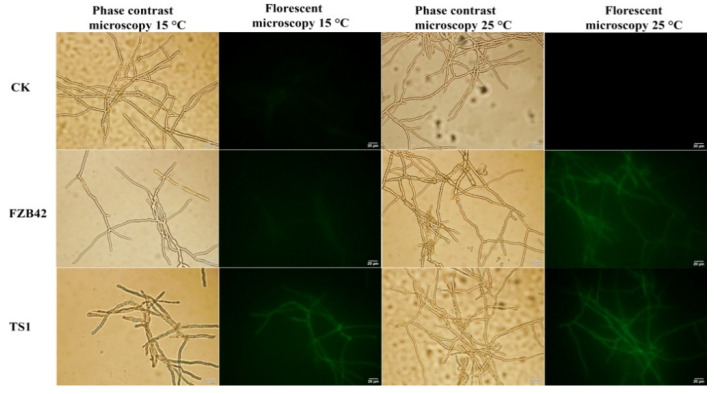
The ROS production in fungal *F. graminearum* hyphae after being treated with *Bacillus* strains FZB42 and TS1 at cold condition 15 °C and normal condition 25 °C. The sterilized LB medium was used to treat the hyphae as a control. The experiment was repeated independently three times.

The fluorescence microscopy results showed that *Bacillus* strains FZB42 and TS1 were able to deteriorate the regular functioning of the fungal cells by inducing the production of reactive oxygen species (ROS) in the *F. graminearum* hyphae. The fungal hyphae treated with FZB42 bacteria showed significant production of ROS at 25 °C as indicated by green fluorescence, whereas the treatment with similar bacteria showed lesser ROS production in fungal hyphae at cold temperature, i.e., 15 °C (Figure 4). The fungal hyphae treated with *Bacillus* strain TS1 showed significant ROS production at 25 °C as well as under cold temperature as indicated by the high intensity of green fluorescence, confirming the potential of this bacteria to induce a disruption in the regular functioning of fungal hyphae at low temperature.

### 2.5. TS1 Induced Ultrastructural Deformities in Fungal Mycelium under Cold Stress

**Figure 5:**
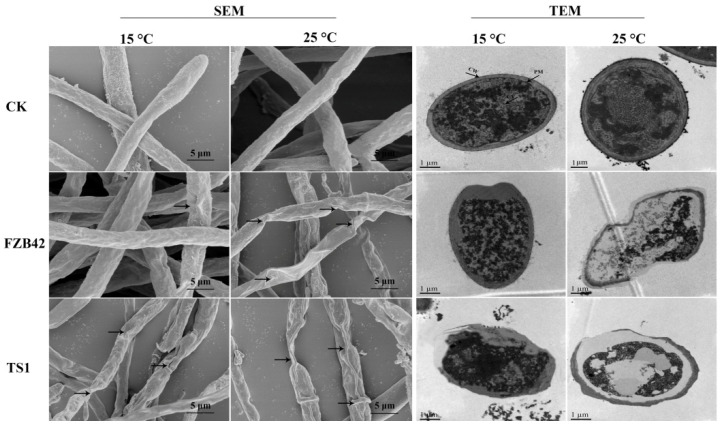
SEM micrographs showing structural deformities in *F. graminearum* hyphae treated with *Bacillus* spp. FZB42 and TS1 at cold condition 15 °C and normal condition 25 °C. Control hyphae are also shown, and the ultra-structural changes inside fungal hyphae are shown by TEM in treated and control samples. CW = cell wall, PM = plasma membrane, and CY = cytosol.

The electron microscopy elucidated the structural deformities in *F. graminearum* caused by the applied *Bacillus* strains. The scanning electron microscope (SEM) micrographs showed surface-level damages in the fungal hyphae. The fungal samples treated with the TS1 strain at 25 and 15 °C showed curling, shrinking, twisting, and plasmolysis in the hyphae as compared to the control hyphae, which were long, dense, and cylindrical, possessing their healthy shape (Figure 5). Conversely, in the FZB42 treated fungal samples, similar deformities were observed at 25 °C, but this strain could not cause major structural damages in fungal hyphae under colder temperatures, i.e., 15 °C.

The ultra-structural changes in fungal hyphae under the treatment of both *Bacillus* strains were also confirmed by transmission electron microscope (TEM). The control/healthy hyphae had good cellular shape and integrity, intact cell membranes, and proper distribution of cytoplasm and organelles. Conversely, the fungal hyphae treated with *Bacillus* strain TS1 showed cellular deformities like loss of cellular integrity, membrane damages, cell shrinkage, cytoplasmic displacement, and degeneration of cellular organelles at 15 °C as well as 25 °C (Figure 5). The TEM results also confirmed similar results to the SEM, as the FZB42 strain induced damages similar to those induced by TS1 in the fungal hyphae at 25 °C, but failed to induce structural deformities in the hyphae of the pathogen at cold temperature.

### 2.6. Phylogenetic Relationship, Motif Composition, and Gene Structure Analysis of Newly Predicted FgHCE and FgNPP1 Gene Families

Phylogenetic trees were constructed by using the MEGA (7.0) for both *HCE* and *NPP1* genes for their domain-based classification with the model fungal genome (*Aspergillus nidulans*). The results demonstrated that the *HCE* phylogenetic tree is subdivided into three different subgroups, as subgroup3 has two *HCE* genes (i.e., *Fusgr_13226* and *Fusgr_12217*), while subgroup1 (*Fusgr_12606*) and subgroup2 (*Fusgr_6199*) only have one gene, respectively. Likewise, the *NPP1* phylogenetic tree is also sub-grouped into subgroup1 (*Fusgr_13018* and *Fusgr_4747*), subgroup2 (*Fusgr_9014*), and subgroup3 (*Fusgr_6975*) (Figure S2).

Moreover, a motif analysis was carried out using the MEME program. The results identified three motifs (motif1–motif3) for *HCE* and four motifs (motif1–motif4) for *NPP1* gene families. Motif1 and motif2 commonly occurred in both (*HCE* and *NPP1*) gene families, signifying the variation in amino acid sequences. A gene structure organization analysis were performed based on untranslated regions (UTRs) and coding sequences (CDS) for both the *HCE* and *NPP1* genes by using TBTools. The results suggested that *HCE* and *NPP1* gene members are highly conserved and exhibit little similarity within subgroups (Figure S3).

### 2.7. Expression Profiling of Fungal Pathogenicity Genes at Cold Temperature

**Figure 6:**
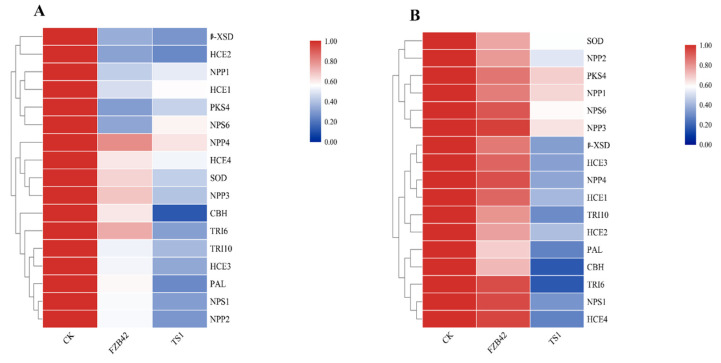
Quantitative real-time PCR expression analysis of pathogenicity-related genes of fungal pathogen *F. graminearum* under treatment of *Bacillus* strains FZB42 and TS1 at (**A**) normal condition 25 °C and (**B**) cold condition 15 °C. The qPCR experiment for expression studies was repeated thrice with similar results.

The results of the transcriptional regulation of pathogenicity-related genes of *F. graminearum* under *Bacillus* treatments at cold temperature exhibited that the *Bacillus* spp. FZB42 and TS1 both significantly downregulated the expression of already reported fungal pathogenicity genes at 25 °C. Both of these bacteria significantly downregulated fungal ROS scavenging genes, i.e., polyphenol ammonia-lyase (*PAL*) and superoxide dismutase (*SOD*), as shown by the heat maps in Figure 6A. The fungal hyphae pre-treated with *Bacillus* strains downregulated the hydrolytic enzymes encoding genes *CBH* (cellobiohydrolase) and β-XSD (β-xylosidase). The genes encoding major fungal mycotoxins such as deoxynivalenol (DON) and zearalenone (ZEN), i.e., *TRI6, TRI10*, and *PKS4*, respectively were also downregulated in the fungal hyphae treated with both bacteria at 25 °C. The *NPS1* and *NPS6* genes encoding the iron scavenging secreted siderophore triacetylfusarinine C (TAFC) were also downregulated by treatment with both *Bacillus* strains at 25 °C. While at 15 °C treatments, only the psychrophilic *Bacillus* strain TS1 was able to significantly downregulate these fungal pathogenicity genes as indicated by the heat maps in Figure 6B. Both strains also showed similar results for two newly predicted gene families, i.e., a necrosis inducing protein gene family (*NPP1, NPP2, NPP3*, and *NPP4*) and a necrosis-related *HCE* gene family (*HCE1, HCE2, HCE3,* and *HCE4*). FZB42 was only able to significantly downregulate the expression of these genes at 25 °C, whereas psychrophilic *Bacillus* TS1 was able to downregulate the expression of all these genes at regular as well as at cold temperature.

### 2.8. In Planta Elicitation of Defense Responses by Inoculated Bacillus at Low Temperature

#### 2.8.1. H_2_O_2_ Accumulation and Callose Deposition

**Figure 7:**
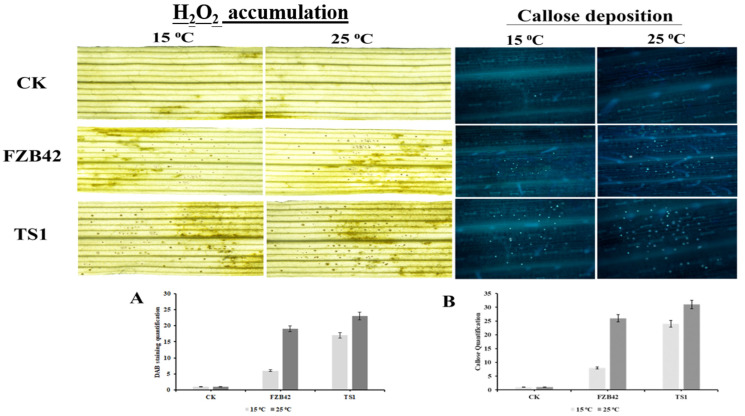
(**A**) The H_2_O_2_ accumulation indicated by brown spots as observed by light microscopy in wheat leaves at 15 and 25 °C; the graph represents the quantification of DAB staining spots. (**B**) The callose deposition spots as observed by fluorescent microscopy in wheat leaves at 15 and 25 °C; the graph represents the quantification of callose deposition spots on wheat leaves. The data in (**A**,**B**) graphs are the mean calculation from 10 different photographs of each treatment. The error bars indicate the standard deviation (SD) from the mean. The significant differences between treatments were taken at *p* ≤ 0.05.

The ability of *Bacillus* spp. FZB42 and TS1 to induce plant defense responses before being challenged with the pathogen showed encouraging results. The wheat leaves inoculated with both strains showed significantly higher accumulation of H_2_O_2_ as compared to the un-inoculated control leaves at 25 °C (Figure 7A). Whereas, at 15 °C, only the psychrophilic TS1 strain was able to induce significant H_2_O_2_ accumulation in the leaves as indicated by dark brown spots on the leaves.

Similarly, at 25 °C, both *Bacillus* strains FZB42 and TS1 showed significant deposition of callose in wheat leaves as compared to the control plants as indicated by intense yellow-greenish spots observed in fluorescent microscopy (Figure 7B). Moreover, the psychrophilic *Bacillus* strain TS1 was able to induce callose deposition significantly in wheat plants under cold temperature, i.e., 15 °C, as well. The results confirmed that FZB42 could not induce both defense responses in wheat plants at 15 °C.

#### 2.8.2. Quantification of Defense Enzymes Activity

**Figure 8:**
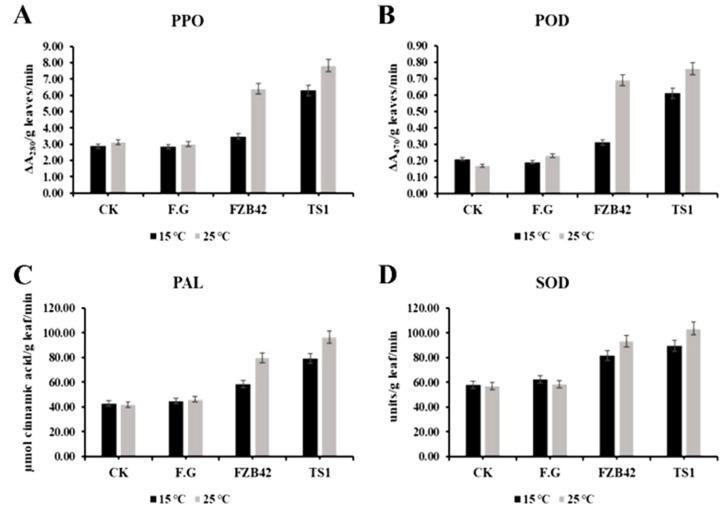
Quantification of defense enzymes activity: (**A**) polyphenol oxidase, (**B**) peroxidase, (**C**) phenylalanine ammonia-lyase, and (**D**) superoxide dismutase in wheat plants under different treatments at 15 and 25 °C. The error bars in the graphs represent the standard deviation (SD) from the means (n = 3). The statistical significance among different treatments was observed at *p* ≤ 0.05 by using Tukey’s HSD test.

The activity of plant defense enzymes under the influence of *Bacillus* spp. FZB42 and TS1 was analyzed at regular and cold temperature. The results showed that the activity of polyphenol oxidase (PPO) was significantly higher in the *B. atrophaeus* strain TS1 at 15 °C as compared to other treatment and controls (Figure 8). At 25 °C, the enzyme activity was found to be significantly higher for both *Bacillus* strains as compared to the controls. Similar patterns of activities of defense enzymes were observed in the case of peroxidase (POD) and phenylalanine ammonia-lyase (*PAL*) with TS1 elucidating the best activity at 15 °C, while at regular temperature, i.e., 25 °C, both strains significantly enhanced enzyme activity. In the case of superoxide dismutase (SOD), both strains were able to positively regulate the enzyme’s activity significantly at regular as well as at cold temperature as compared to the healthy and infected controls.

#### 2.8.3. Expression Profiling of Plant Defense Responsive Genes

**Figure 9:**
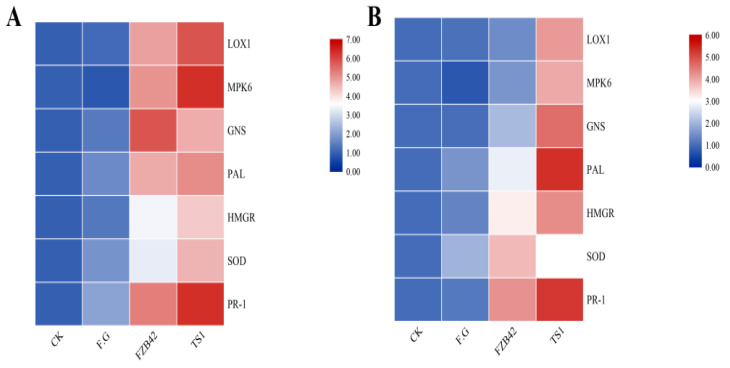
Quantitative real-time PCR expression profiling of defense-related genes in wheat under influence of *Bacillus* spp. at (**A**) normal 25 °C and (**B**) cold 15 °C. The expression study was repeated three times with similar results.

The expression analysis of plant defense-related genes in wheat showed that both *Bacillus* strains FZB42 and TS1 were able to modulate the transcriptional regulation of these genes. The *Bacillus* strains FZB42 and TS1 upregulated the expression of all genes under study in the presence of *F. graminearum* at 25 °C as compared to the healthy control as well as pathogen-challenged control (Figure 9A), whereas most of the genes were downregulated in plants challenged only with the fungal pathogen. The maximum expression was observed for the lipoxygenase encoding gene (*LOX1*), mitogen-activated protein kinase (*MPK6*), and pathogenicity-related gene (*PR1*) in wheat plants treated with TS1 strain in the presence of pathogen. The GNS encoding the Endoglucanase gene in wheat was most upregulated in FZB42 treated samples at 25 °C. At cold temperature, the TS1 strain showed significantly higher expression of all defense-related genes in the plants as compared to all other treatments with maximum expression observed for pathogenicity-related gene (*PR1*) and Phenylalanine ammonialyase gene (*PAL*) (Figure 9B). Whereas, at 15 °C the *Bacillus* strain FZB42 showed just slight upregulation of defense-related genes as compared to the control treatments.

#### 2.8.4. Diseased Leaf Area

**Figure 10:**
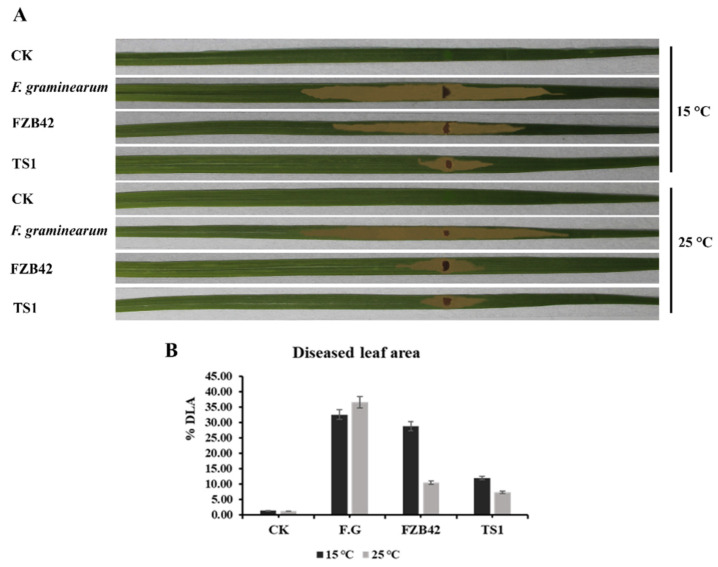
(**A**) The lesions on the leaves showed diseased leaf area in different wheat treatments at 15 and 25 °C. (**B**) The graph shows % diseased leaf area in wheat under different treatments at 15 °C and 25 °C. The standard error of the means is represented by the error bars on the graphs. The statistical differences among different treatments were calculated by Tukey’s HSD test at *p* ≤ 0.05.

The diseased leaf area percentage (% DLA) in pathogen-challenged wheat plants inoculated with *Bacillus* spp. was observed to be significantly reduced (Figure 10A). At 15 °C, the psychrophilic *Bacillus* strain TS1 reduced the % DLA to 11.9% as compared to the 32.4% of the infected control (Figure 10B). The FZB42 treated plants did not show a significant reduction in lesion lengths as compared to the infected control. Whereas, at 25 °C, both strains FZB42 and TS1 significantly reduced the % DLA from 36.4% (infected control plants) to 10.4% and 7.1%, respectively, in *Bacillus* inoculated plants. The psychrophilic strain TS1 was shown to significantly reduce the % DLA at regular as well as at low temperature.

## 3. Discussion

*Bacillus* spp. have been reported to alleviate biotic and abiotic stresses in plants by modulating their phytohormones and elicitation of plant defense responses [27,28,29]. The ability of these bacteria to produce extracellular hydrolytic enzymes and lipopeptides (LPs) through non-ribosomal protein synthetases (NRPS) enzyme complexes contributes directly towards the suppression of phytopathogens [30,31]. These rhizospheric *Bacillus* strains can also stimulate the plant defense response. Recent studies have considered some lipopeptides as the latest class of microbe-associated molecular patterns (MAMPS) and elicitors of the plant immune response via triggering ISR [32,33]. The present study elaborates the ability of the psychrophilic *B. atrophaeus* strain TS1 to induce structural, functional, and novel genetic dysregulations in *F. graminearum* under cold environments. The bacteria were also able to elicit a defense response in wheat plants at cold temperatures, hence reducing the disease severity.

The extreme habitat of the Qinghai-Tibetan region has instilled the *B. atrophaeus* strain TS1 with the ability to withstand harsh environmental conditions while performing its physiological and metabolic functions efficiently. This study presents a comparative analysis of *Bacillus* TS1 and model temperate *B. amyloliquefaciens* strain FZB42, which has been extensively reported for its biocontrol ability at optimal temperatures, to suppress the fungal pathogen and control plant diseases at optimal as well as cold temperature [34,35]. The TS1 strain could inhibit the growth of *F. graminearum* more effectively as compared to FZB42 at low temperature as indicated by the higher inhibition diameter and 21% suppression of conidial germination rate (SCGR %), and similar results have also been reported where *B. amyloliquefaciens* suppressed the conidial germination of *Fusarium oxysporum* [36]. The elevated production of extracellular enzymes by the biocontrol *Bacillus* spp. has also been considered to be effective in suppressing the fungal pathogens *P. lycopersici* and *F. graminearum* [37,38]. Our findings have shown a significant increase in the production of hydrolytic extra-cellular enzymes (lipase, amylase, cellulase, and protease) by the TS1 strain as compared to FZB42 at 15 °C, and this could contribute to increased pathogen inhibition at the lower temperature. The role of iron-chelating compounds siderophores has been well reported for the control of plant pathogens [39], and, as the TS1 strain also possesses genetic machinery for the production of a major functional class of siderophores, i.e., pyoverdin [40], we assume this can also contribute towards the biocontrol potential of this psychrophilic bacteria at low temperature.

The genome of *Bacillus* spp. contains genetic features responsible for producing a repertoire of antimicrobial lipopeptides (LPs). These lipopeptides, i.e., surfactin, bacillomycin, and fengycin, are frequently reported to suppress phytopathogens [41,42]. The current study evaluates the comparative quantification of LPs, i.e., surfactin, bacillomycin, and fengycin, through LC-MS at optimal growth temperature (25 °C) and cold temperature (15 °C). The psychrophilic strain TS1 is able to grow and produce metabolites adequately at low temperatures [26]. It was able to produce higher quantities of these LPs at both temperatures as compared to the temperate strain FZB42, which could not produce higher quantities of these LPs at a lower temperature as indicated by the peak area and the intensity of individual peaks (Table S1). The corresponding results were attained by the transcriptional regulation of genes responsible for the biosynthesis of bacillomycin (*bacy*), fengycin (*feng*), and surfactin (*srfa*) in FZB42 and TS1 strains at optimal and cold temperature. Many studies have attributed the suppression of phytopathogenic fungi such as *Aspergillus nidulans*, *F. graminearum*, and *Verticillium dahliae* to the LPs produced by the inoculated *Bacillus* spp [43,44,45], but not many have evaluated the production of LPs and subsequent suppression of *F. graminearum* at low temperature.

The adequate production of LPs by the TS1 strain at lower temperature was crucial in inducing structural and functional dysregulations in *F. graminearum* at low temperature. Both strains FZB42 and TS1 were able to induce structural deformities, namely, plasmolysis, curling, shrinkage, and pore formation as observed by SEM in the fungal hyphae at regular temperature, while TEM also showed similar membrane and intra-cellular damages. There have been many studies indicating the structural damage of fungal hyphae by the interaction of LPs with sterol and phospholipid molecules of the fungal cell membranes [46,47]. However, very few studies have reported the ultra-structural deformities by the action of the *Bacillus* strain at low temperature. Further evidence for the suppression of fungi by the psychrophilic strain TS1 was provided through its ability to induce oxidative damage in the fungal hyphae at a lower temperature as indicated by a higher accumulation of reactive oxygen species (ROS). ROS produced in higher amounts cause cell damages leading to cell death [48], and many studies have reported the oxidative damage in fungal cells of *Rhizopus stolonifer* and *F. graminearum* by the inoculated *Bacillus* spp. [12,49]. The expression analysis of ROS scavenging enzymes in *F. graminearum*, i.e., superoxide dismutase (*SOD*) and Phenylalanine ammonia-lyase (*PAL*), also showed a downregulation under the treatment of both *Bacillus* strains at 25 °C and specifically after treatment with TS1 at 15 °C, further strengthening the claim of oxidative damage in pathogenic fungus *F. graminearum* by TS1 at low temperature.

Multiple studies have indicated downregulation in the expression of the pathogenicity-linked genes of phytopathogens after the treatment with *Bacillus* species or their secondary metabolites, namely, in *Magnoporthe grisea* and *F. graminearum* under treatment of *B. subtilis* and *B. amyloliquefaciens*, respectively [15,50]. The genome-wide analysis of *F. graminearum* was conducted for predicting novel necrosis-inducing protein families involved in causing necrosis in host plants and the current study presents precise identification, structural/motif prediction, and expression profiling of eight unique genes belonging to *HCE* (PF14856.6; pathogen effectors; putative necrosis-inducing factor) and *NPP1* (PF05630.11; necrosis-inducing protein) families. The expression analysis of these newly predicted gene families depicted downregulation in the transcript levels of *HCE1*, *HCE2*, *HCE3*, *HCE4* and *NPP1-1*, *NPP1-2*, *NPP1-3*, *NPP1-4* genes from the *Bacillus* treated hyphae with maximum downregulation at 15 °C observed in TS1-treated hyphae. To the best of our knowledge, the involvement of these genes against fungal infection in plants has not been reported yet in any of the previous studies. The present work also reports the expression analysis of pathogenicity-linked genes in fungus, at low temperature, already reported to be downregulated under the influence of inoculated bacteria. *TR16*, *TRI10*, and *PKS4* genes involved in the production of mycotoxins by *F. graminearum*, such as deoxynivalenol (DON) and zearalenone (ZEN), respectively [51,52], had significantly lower expression in the fungal hyphae treated with TS1 at low temperature. The *F. graminearum* genes responsible for the production of extracellular siderophores such as TAFC and malonichrome have a major role in iron transport leading to oxidative stress regulation in pathosystems [53]. The *Bacillus* strains FZB42 and TS1 were able to downregulate the corresponding *NPS6* and *NPS1* fungal genes at regular temperature, whereas TS1 also induced significant downregulation at low temperature; these results serve as further evidence of the reduction in fungal virulence due to downregulation of these genes, and the results are consistent with those of previous studies [54,55]. A similar trend of expression downregulation was observed in the case of *β-XSD* and *CBH* genes involved in the biosynthesis of *F. graminearum* hydrolytic enzymes β-xylosidase and cellobiohydrolase, respectively, involved in degrading the plant defensive machinery [56].

In addition to the downregulation of pathogenicity-linked genes in *F. graminearum*, the *Bacillus* spp. and their products are reported to be stimulators of the plant immune response and induced systemic resistance (ISR) [57,58]. The sudden accumulation of H_2_O_2_ and deposition of callose are considered to be important cellular plant defense signals against phytopathogens [59]. At 25 °C, the inoculated *Bacillus* spp. FZB42 and TS1 were able to induce these hallmarks of the plant defense response, preparing the plant for defense even before the pathogen inoculation in accordance with a previous study showing the elicitation of the defense response by inoculated *B. subtilis* against *B. cinerea* infection [60]. TS1 inoculated plants had the sudden significant induction of these defense-related molecules in wheat plants as compared to FZB42 at low temperature as well. Similar results were obtained in wheat plants treated with *Bacillus* spp. FZB42 and TS1 for the induction of defense-related enzymes, i.e., polyphenol oxidase (*PPO*), peroxidase (*POD*), phenylalanine ammonia-lyase (*PAL*), and superoxide dismutase (*SOD*). In parallel to our results, many studies have focused on the role of PPO and POD in the oxidation of phenols and plant disease resistance [61,62]. The increase in the enzyme activity of SOD and PAL in our study shows the ability of *Bacillus* spp. to protect the plants against the adverse effects of phytopathogens. Our results are supported by previous studies stating SOD as a major cellular protectant against oxidative burst in plants against phytopathogens [63], while PAL is reported to be involved in the production of antimicrobials such as lignins, coumarins, and flavonoids in plants by catalyzing the phenylpropanoids [64]. The wheat plants treated with FZB42 and TS1 showing higher enzyme activities for SOD and PAL corresponded to the higher expression of their encoding genes in *F. graminearum* challenged plants at optimal plant growth temperature and solely in TS1-treated plants at low temperature.

The ability of *Bacillus* spp. to increase the activity of defense enzymes coupled with their potential to upregulate the expression of defense-related genes in plants serves as a powerful tool for the protection of plants from phytopathogens [65,66]. Both of the inoculated strains FZB42 and TS1 were able to enhance the expression of defense-related genes (*LOX1*, *MPK6*, *GNS*, *HMGR*, and *PR-1*) in wheat plants challenged with a pathogen at 25 °C. Whereas, in plants at 15 °C, only TS1 was able to enhance the expression of these genes significantly. *LOX1* is involved in the biosynthesis of oxylipins able to regulate JA/SA synthesis, and a higher expression of *LOX1* leads to an increased defense response against *F. graminearum*, similar to the case in our study [67]. The over-expression of *MPK6* and *GNS* genes in our study corresponds to the previous study as these are involved in the activation of mitogen-activated protein kinases signaling acting as the first line of defense for plants against fungal infection [68] and the production of hydrolytic enzyme β-1,3 endoglucanase, respectively, helping the wheat plants have enhanced resistance to pathogen attack [69]. The increased expression of the HMG-CoA reductase enzyme encoding gene *HMGR* and the pathogenesis-related gene *PR-1* of wheat in the current study is parallel to previous studies describing the role of these genes in catalyzing the isoprenoid regulated and general pathogen response mechanisms in plants, respectively, leading to a significantly enhanced plant defense response against *B. cinerea* [70,71]. The orchestrated effect of inoculated *Bacillus* sp. in the accumulation of H_2_O_2_ and the deposition of callose as plant defense regulators acts as the first line of defense in preparing the plants for the pathogen attack. The *in planta* experiment at 15 °C and 25 °C also revealed significant increase in the activity of key oxidative enzymes involved in plant defense under the treatment of inoculated bacteria coupled with the higher expression of defense-linked genes in wheat. These phenomena led to the elicitation of an effective plant immune response against *F. graminearum* and resulted in a significant decrease of diseased leaf area (% DLA) in the wheat plants.

## 4. Material and Methods

### 4.1. Growth Conditions of the Fungal Pathogen and Bacillus spp.

The previously isolated cold-tolerant strain *B. atrophaeus* TS1 and cold non-tolerant biocontrol *B. amyloliquefaciens* strain FZB42 were procured from the Lab of Biocontrol and Bacterial Molecular Biology, Nanjing Agricultural University, Nanjing, P.R China. The strains were preserved in Luria-Bertani (LB) broth media amended with 40% (*v*/*v*) glycerol and stored at −80 °C. The bacterial cultures were refreshed by being grown at 37 °C for 24 h before use in all experiments. The fungal pathogen *F. graminearum* PH-1 strain (lab preserved) was patched onto potato dextrose agar (PDA) media and incubated at 25 °C for 4 days before use. All the microbes were grown in the respective medium at 15 °C and 25 °C for testing cold tolerant and non-tolerant characteristics.

### 4.2. In Vitro Fungal Inhibition at Low Temperature

#### 4.2.1. Dual Culture Assay for Antagonism

The dual culture test was performed to check the antagonistic activity of *Bacillus* strains TS1 and FZB42 against *F. graminearum* at regular as well as in cold environment. A mycelial plug of 0.6 cm from fungus grown for 4 days was patched in the center of PDA medium plates and 5 µL of each bacterial suspension with optical density (OD) of 2.50 (at 600 nm) was inoculated 3 cm away from the patched mycelium. Sterilized LB media was also used in the same plate as control [7]. The plates were incubated at 25 °C and 15 °C for 4 days to observe the fungal inhibition zone at optimal growth temperature as well as at cold conditions.

#### 4.2.2. Conidial Germination Suppression

The conidia of *F. graminearum* were collected by growing the fungus on PDA plates for 4 days at 25 °C by adding sterilized double distilled water (ddH_2_O) on the plate with constant stirring; subsequently, the liquid was filtered to remove mycelia by using degreasing cotton. One mL of conidial suspension (10^4^/mL) was transferred to microcentrifuge tubes containing liquid cultures of *Bacillus* strains FZB42 and TS1 grown at 15 and 25 °C separately. The tubes were incubated at room temperature for treatment with bacteria for 2 h. The tubes were then centrifuged at 10,000 rpm for 15 min (min) at room temperature followed by two washings with ddH_2_O. The conidia were then re-suspended in potato dextrose broth (PDB) for 4 h at 15 and 25 °C. The conidial germination rate at normal and low temperature was observed and calculated by using optical microscopy. The percentage for the suppression of conidial germination rate (SCGR %) was estimated by using the formula reported previously [37].

#### 4.2.3. Screening for Biocontrol Determinants under Cold Stress

The *Bacillus* strains FZB42 and TS1 were evaluated for their ability to produce extracellular enzymes and siderophores at regular growth temperature, i.e., 25 °C, and under cold stress, i.e., 15 °C. Lipase production was detected by using Tween-20 supplemented peptone agar media as described by [72]. The amylase production was checked by inoculating the bacteria on nutrient agar (NA) plates amended with 2% soluble starch and were then overlaid with iodine solution [73]. Siderophores production was assessed by using chrome azurol S (CAS) medium as described by [74] at a low and regular temperature. The production of protease and cellulase was detected on skimmed milk agar media [75] and NA amended with 2% carboxymethylcellulose (CMC), subsequently overlaid with 0.1% Congo red solution [76]. The clear zones around the bacterial colonies showed the production of the lytic enzymes. The plates with both *Bacillus* strains were incubated at 15 and 25 °C separately for 72–96 h.

### 4.3. Molecular Detection of Genes Encoding Extracellular Enzymes and Siderophores

The genome of cold-tolerant biocontrol *B. atrophaeus* strain TS1 was screened for the presence of genes responsible for the production of extracellular enzymes and siderophores. The genomic DNA was extracted from bacterial cells harvested from 24 h of grown culture in LB medium. A Bacterial DNA Extraction Kit (Omega Bio-tek, Norcross, GA, USA) was used to extract DNA by using the manufacturer’s guidelines. The gene sequences from *Bacillus* model strain 168 were retrieved from NCBI and searched in the TS1 genome by using local alignment tools. The primers were synthesized using the PrimerQuest tool of Integrated DNA Technologies (IDT). A DNA Master Mix (Vazyme Biotech. Co. Ltd., Nanjing, China) was used to amplify the targeted genes by using the reaction mixture and PCR profile provided by the manufacturer. The PCR primers used in this are listed in Table S2.

### 4.4. Liquid Chromatography-Mass Spectrometry (LC-MS) Analysis under Low Temperature

The antimicrobial lipopeptides (LPs) were detected at 25 and 15 °C for evaluating the ability of the cold-tolerant TS1 strain and cold-sensitive FZB42 strain to produce these compounds under cold stress. For LC-MS analysis, the *Bacillus* strains were grown in Landy medium for 72 h at 25 and 15 °C in a shaking incubator. The cultures were centrifuged at 10,000 rpm for 10 min at 4 °C to obtain cell-free supernatant and the samples were left at room temperature for 24 h after adjusting the pH to 2 by using 3 M hydrochloric acid (HCl). The precipitates were collected by centrifugation under the same conditions and the samples were then dissolved into 5 mL of methanol. All samples were filter sterilized by using 0.2 µM syringe filters [77].

The intensity of antimicrobial LPs was detected by a surveyor LC-MS-system (G2 QT of-XS, Waters). The separations were done by using a UPLC C18 column (2.1 × 100 mm) with Acquity UPLC BEH particles. An amount of 2 µL of sample was injected into the machine having with electrospray ionization (ESI) in positive ion mode [M+H] + with MSE acquisition in the range of 50–1200 *m*/*z*. A similar run phase and protocol were used as already reported by our lab [78]. The data collection and analysis were done using Mass Lynx software (Version 4.1) (https://www.waters.com/waters/en_US/MassLynx-Mass-Spectrometry-Software-/nav.htm?cid=513164&locale=en_US accessed on 2 November 2019).

### 4.5. Expression Analysis of Lipopeptide Biosynthetic Genes under Cold Stress

To analyze the expression of lipopeptide biosynthetic genes (fengycin, surfactin, and bacillomycin), total RNA was extracted from harvested cells of bacterial strains FZB42 and TS1 grown in LB medium at 15 and 25 °C. RNA was extracted using a Bacterial RNA Extraction Kit (OMEGA Bio-tek, Norcross, GA, USA) by following the manufacturer’s instructions. The extracted RNA with known concentration and purity was used to synthesize cDNA by using a 5X All-In-One RT MasterMix kit (abm^®^, Beijing, China) as instructed by the manufacturer.

The relative expression of LP biosynthetic genes was quantified by using SYBR Green Pre-mix Ex-Taq (Takara Bio, Beijing, China) with ROX as a reference dye. The reaction mixture was prepared according to the manufacturer’s guidelines. Quantitative PCR was carried out in a QuantStudio 6 Flex Real-time PCR System with 20 µL reaction volume. The primers for LP genes were designed by using the PrimerQuest tool by IDT after retrieving gene sequences from NCBI and are listed in Table S2. The *rpsj* gene was taken as a house-keeping endogenous control for *Bacillus* as previously reported [79]. The final relative change in expression of the targeted genes was calculated by using the comparative CT method of 2^−ΔΔCT^ as previously described by [80].

### 4.6. Bacillus Induced ROS Production in F. graminearum under Low Temperature

The production of reactive oxygen species (ROS) in the *F. graminearum* hyphae was observed after treating them with bacterial culture suspensions. To assess the potential of biocontrol *Bacillus* spp. FZB42 and TS1 to retard the cellular functioning of the fungus under cold stress, the fungus and bacteria were grown separately using their respective media at 15 and 25 °C. The hyphae were treated with the bacterial cultures for 12 h at regular as well as cold temperature and sterilized LB medium was used to treat the hyphae in control samples. The hyphae were then centrifuged at 10,000 rpm for 8 min and the collected hyphae were re-suspended in 10 mM sodium phosphate buffer (pH 7.4). The hyphae were incubated at 37 °C with 10 µM of probe dye dichloro-dihydro-fluorescein diacetate (DCFH-DA) which comes with an ROS staining kit (JianCheng Bioengineering, Nanjing, China). This dye can label the cells producing ROS and a green fluorescence can be detected by using a fluorescent microscope (Olympus IX71, Tokyo, Japan) [81]. The experiment was repeated three times to confirm the results.

### 4.7. Ultrastructural Deformities in Fungal Mycelium under Cold Stress

The fungal mycelium was observed for ultrastructural changes under the influence of biocontrol *Bacillus* strain FZB42 and TS1 by using electron microscopy. The *F. graminearum* hyphae were treated with bacterial cultures and incubated for 24 h at 15 and 25 °C. The fungal hyphae treated with LB medium served as control. The treatment was followed by the fixation of fungal hyphae with a 2.5% glutaraldehyde solution. Then, 100 mM phosphate buffer was used to rinse the fixed fungal hyphae for 10 min, and this was repeated three times. Subsequently, the hyphae were fixed again in 1% osmium tetroxide and dehydrated by incubations with an ethanol gradient as described previously [12]. Then, the samples were gold-coated and analyzed for surface deformities by using a scanning electron microscope (Hitachi S-3000N, Tokyo, Japan). Transmission electron microscope (Hitachi H-600, Tokyo, Japan) was used to observe structural damage inside the fungal cells after embedding the samples in Epon 812 and sectioning with an ultra-microtome.

### 4.8. Genome-Wide Analysis of Novel Necrosis-Inducing Gene Families in F. graminearum

#### 4.8.1. Gene Mining and Identification for *HCE* and *NPP1*

The unique gene families were searched using the pfam IDs obtained from the pfam database (https://pfam.xfam.org/ accessed on 2 November 2019) for both *HCE* (PF14856.6; pathogen effector; putative necrosis-inducing factor) and *NPP1* (PF05630.11; necrosis-inducing protein). The genomic sequences of *HCE* and *NPP1* were retrieved/searched from the *F. graminearum* genome (https://www.ncbi.nlm.nih.gov/nuccore/758213871?report=genbank/ accessed on 2 November 2019) in comparison to the *Aspergillus nidulans* genome (https://www.ncbi.nlm.nih.gov/nuccore/50058547?report=genbank/ accessed on 2 November 2019), which was used as the model fungus for this study. The retrieved sequences were further verified for *HCE* and *NPP1* domains using the website NCBI-Conserved Domain database (https://www.ncbi.nlm.nih.gov/Structure/cdd/wrpsb.cgi/ accessed on 2 November 2019). Moreover, the sequences with domain errors and shorter nucleotide length (<100 bp) were removed before further analysis.

#### 4.8.2. Phylogenetic Analysis of *HCE* and *NPP1*

The amino acid sequences of *HCE* and *NPP1* gene families were aligned using MUSCLE integrated into MEGA 7.0 software. The same software was used to construct the phylogenetic trees using the maximum likelihood (ML) method. For determining the reliability of the resulting phylogenetic trees, bootstrap values of 1000 replications were evaluated with the Jones, Taylor, and Thornton amino acid substitution model (JTT model) as described previously [82].

#### 4.8.3. Gene Structure, Conserved Motifs Analysis, and Physicochemical Parameters of *HCE* and *NPP1* Proteins

The gene structure was elaborated by using TBtools software through the utilization of the GFF3 file of the *F. graminearum* genome as described previously [83]. The scanning for conserved motifs constituted in *HCE* and *NPP1* proteins was carried out through local MEME Suite (Version 5.0.5) and then visualized through TBtools software. The parameter settings calibrated for the same purpose were set as maximum number of 10 motifs and size ranging in between width of 50 and 100. The physicochemical properties of the newly predicted *HCE* and *NPP1* proteins (i.e., molecular weight (MW), isoelectronic points (PIs), were determined using the ExPASY PROTPARAM tool (http://web.expasy.org/protparam/ accessed on 2 November 2019).

#### 4.8.4. Expression Profiling of Bacillus-Treated Fungal Pathogenicity Genes under Cold Stress

The gene expressions of fungal pathogenicity-linked genes as identified through genome-wide analysis and those involved in mycotoxins production were analyzed. The mycelia were harvested from *F. graminearum* grown in PDB medium and treated for 12 h with the cultures of *Bacillus* strains FZB42 and TS1 grown at 15 and 25 °C in LB medium. The mycelia treated with only LB medium served as control. Total RNA was extracted after grinding the mycelia in liquid nitrogen and using the Takara RNAiso Reagent Kit (Takara Bio, Beijing, China) by following the manufacturer’s guidelines. The cDNA synthesis and qPCR were performed similarly as described in Section 2.6, with actin being used as the housekeeping gene. The qPCR primers for the expression analysis of the fungal pathogenicity-linked genes used in this study are listed in Table S2.

### 4.9. In Planta Assays for Bio-Control by Cold-Tolerant Bacillus Strain

The antagonistic potential of *Bacillus* spp. FZB42 and TS1 against *F. graminearum* pathogen was assessed *in planta* at regular as well as cold temperatures. The wheat seeds of the cultivar Jimai22 were surface-sterilized by using 5% sodium hypochlorite solution followed by washing with 70% ethanol and subsequent two washings with ddH_2_O. The seed priming was done independently with *Bacillus* spp. FZB42 and TS1 by dipping the seeds in bacterial cells (OD_600_ = 2 and 10^7^ CFU/mL) suspended in ddH_2_O for 30 min after harvesting from LB media grown overnight. The control (healthy and infected) seeds were dipped in sterilized water only. The plastic pots containing compost: vermiculate (30:70) were used to sow five seeds per pot and nine pots for each treatment. The pots were incubated separately at 15 and 25 °C in growth chambers having a 16 h/8 h light/dark cycle.

#### 4.9.1. Induction of Defense Response

The *Bacillus* strains were tested for their ability to induce a defense response in plants before the pathogen inoculation. It was examined in wheat plantlets grown at regular (25 °C) as well as cold temperature (15 °C) by spraying the already primed and healthy control plants with a bacterial suspension (10^9^ CFU mL^−1^) of *Bacillus* spp. FZB42 and TS1 on the 21st day, whereas the spray of ddH_2_O was used as control. The plants were checked for induction of defense responses 24 h post-spraying.

Diaminobenzidine (DAB) staining was performed for checking the accumulation of hydrogen peroxide (H_2_O_2_) in wheat leaves cut from each treatment by following a previously described method [84]. Diaminobenzidine (DAB) (Sigma, St. Louis, MO, USA) (1 mg/mL, pH 3.8) was used to stain the leaves at room temperature for 8 h. The leaves were then boiled by using ethanol 96% (*v*/*v*) for 10 min and were subsequently preserved in 50% ethanol. Light microscopy was used to visualize dark brown spots on the leaves showing the accumulation of H_2_O_2_. The captured images were used to calculate the DAB intensity by considering the brown pixels as already reported [85].

Callose deposition was examined by fixing the leaves from each treatment for 4 h in 3:1 ethanol: acetic acid solution [86]. Following the boiling of the leaves at 60 °C for 20–30 min for clearing chlorophyll, the leaves were soaked in 3 mL of 0.01% aniline blue solution containing 150 mM dipotassium hydrogen phosphate (K_2_HPO_4_) and incubated in the dark for 2–4 h. The callose deposition was observed by a fluorescence microscope under UV light and analyzed for quantification by using ImageJ software as previously described [87].

#### 4.9.2. Quantification of Defense Enzymes

The defense enzymes were quantified from the plant leaves from all treatments grown at 15 and 25 °C. Three leaves were harvested and mixed independently from pots of each treatment 48 h post-pathogen inoculation. For the quantification of different defense enzymes, 0.1 g fresh weight of leaves was used for each sample. For the extraction of polyphenol oxidase (PPO), L-tyrosine was used as substrate in the enzymes assay and quantified by using a spectrophotometer by measuring the OD_600_ at 280 nm as described previously [88]. Guaiacol was used as a substrate to assay the activity of peroxidase (POD) at 470 nm wavelength [89]. The activity of phenylalanine ammonia-lyase (PAL) was measured by determining the conversion of L-phenylalanine to trans-cinnamic acid using a spectrophotometer at 290 nm wavelength [90]. The superoxide dismutase (SOD) activity was measured by using the nitroblue tetrazolium (NBT) procedure previously described [91]. One unit of SOD was defined as the amount of SOD needed for 50% inhibition of NBT reduction measured at 560 nm using a spectrophotometer.

#### 4.9.3. Expression Profiling of Plant Defense Responsive Genes

The expression analysis was done for all treatments to quantify the expression of plant defense-related genes in response to *Bacillus* treatment at 15 and 25 °C. The leaves from each treatment were harvested at 48 h post-pathogen inoculation and ground to a fine powder in liquid nitrogen. The total plant RNA was extracted by using a Plant RNA Extraction Kit (OMEGA Bio-Tek, Norcross, GA, USA) by following the manufacturer’s instructions. The cDNA synthesis and qPCR reactions were carried out by using the same protocols and reaction conditions as described earlier. The genes studied for transcriptional regulation during the plant defense response to the pathogen were *LOX1* (lipoxygenase), *MPK6* (mitogen-activated protein kinase), *gns* (beta-1,3-endoglucanase), *PAL* (phenylalanine ammonia-lyase), *HMGR* (3-hydroxy-3-methylglutaryl co-A reductase), *SOD* (superoxide dismutase), and *PR-1* (pathogenicity-related gene). Actin was used as an endogenous control gene. The primers used for evaluating the expression of the wheat plants are listed in Table S2.

#### 4.9.4. Diseased Leaf Area

The wheat leaves were inoculated with *F. graminearum* after 24 h of foliar spray of the bacterial suspension. The fungus was grown on a PDA plate at 25 °C for 4 days and then a mycelial plug of 0.6 cm was placed on the leaf surface and wrapped with para-film. The fungus was inoculated in pots of infected control (pathogen only) and plants treated with *Bacillus* strains, whereas healthy control plants were treated with a 0.6 cm plug of PDA medium only and wrapped with parafilm. The disease symptoms were observed in the form of chlorotic lesions on the leaves in all pathogen-challenged treatments. Percent diseased leaf area (% DLA) was measured seven days’ post-pathogen inoculation by using the following formula:

% DLA = Total lesion length of leaf/total length of leaf × 100(1)

The % DLA was calculated for seven pots of five plants each from all treatments. The experiment was repeated in triplicate to confirm the results.

### 4.10. Statistical Analysis of Data

Completely randomized design was used to conduct all the in vitro and in vivo experiments in the present study, with each experiment repeated thrice. The statistical analysis was performed by using the statistical package SPSS. Tukey’s HSD test was applied for the separation of means at *p* ≤ 0.05 after conducting the analysis of variance (ANOVA) for all data sets.

## 5. Conclusions

This study was focused on evaluating the biochemical and transcriptional potential of the psychrophilic *B. atrophaeus* strain TS1 to produce biocontrol determinant enzymes and lipopeptides (surfactin, bacillomycin, and fengycin) for the suppression of phytopathogen *F. graminearum* at low temperature. The *Bacillus* sp. caused oxidative damage, structural deformities, and expressional disregularities in novel pathogenicity-linked fungal genes, predicted eight novel genes potentially belonging to necrosis-inducing protein families *fgHCE* and *fgNPP1* by genome-wide analysis of the fungus. The inoculated *Bacillus* strain TS1 also triggered the plant immune response against the fungal pathogen at regular as well as at low temperature. In addition to offering novel avenues to investigate the molecular basis of *F. graminearum* pathogenesis, the present study also provides valuable insights into the use of TS1 as a potential bio-pesticide for plant disease control under cold environments.

## Data Availability

Not applicable.

## Supplementary Materials

The following are available online at https://www.mdpi.com/1422-0067/22/22/12094/s1.
