# Supplementary material for: Novel Genetic Dysregulations and Oxidative Damage in Fusarium graminearum Induced by Plant Defense Eliciting Psychrophilic Bacillus atrophaeus TS1
Source: Int J Mol Sci. 2021 Nov 9;22(22):12094. doi: 10.3390/ijms222212094 (PMC8622878; doi:10.3390/ijms222212094)
Supplement: Supplementary file 1 [file ijms-22-12094-s001.zip › ijms-1413044-supplementary.pdf]

International Journal of *Molecular Sciences*

MDPI

Supplementary data file:

Novel Genetic Dysregulations and Oxidative Damage in *Fusarium graminearum* Induced by Plant Defense Eliciting Psychrophilic *Bacillus atrophaeus* TS1

Muhammad Zubair [1], Ayaz Farzand [1,2], Faiza Mumtaz [3], Abdur Rashid Khan [1], Taha Majid Mahmood Sheikh [1], Muhammad Salman Haider [4], Chenjie Yu [1], Yujie Wang [1], Muhammad Ayaz [1], Qin Gu [1], Xuewen Gao [1] and Huijun Wu [1,*]

[1] Department of Plant Pathology, College of Plant Protection, Nanjing Agricultural University, Key Laboratory of Integrated Management of Crop Diseases and Pests, Ministry of Education, Nanjing 210095, China; zubair_biotech@yahoo.com (M.Z.); ayaz.farzand@uaf.edu.pk (A.F.); malix.477@gmail.com (A.R.K.); tahamajid1705@yahoo.com (T.M.M.S.); yuchenjie0501@163.com (C.Y.); 2019202005@njau.edu.cn (Y.W.); m.ayazbiotech@gmail.com (M.A.); guqin@njau.edu.cn (Q.G.); gaoxw@njau.edu.cn (X.G.).

[2] Department of Plant Pathology, University of Agriculture, Faisalabad 38040, Pakistan

[3] Department of Pharmacology, School of Medicine, Tehran University of Medical Sciences, P.O. Box 13145-784,Tehran, Iran; mumtaz.faiza@yahoo.com.

[4] College of Horticulture, Nanjing Agricultural University, Nanjing 210095, China; salman.hort1@gmail.com.

* Correspondence: hjwu@njau.edu.cn; Tel. +86-25-84395268; Fax +86-25-84395268.

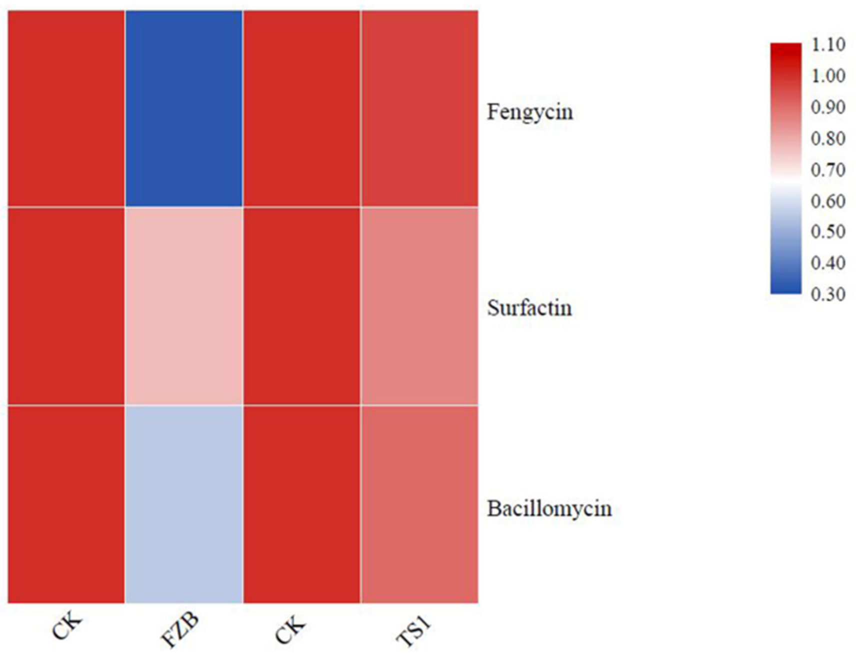

Figure S1. The heat map represents the expression profiling of LPs encoding genes in *Bacillus* spp. under cold temperature. The expression study was repeated thrice with similar results.

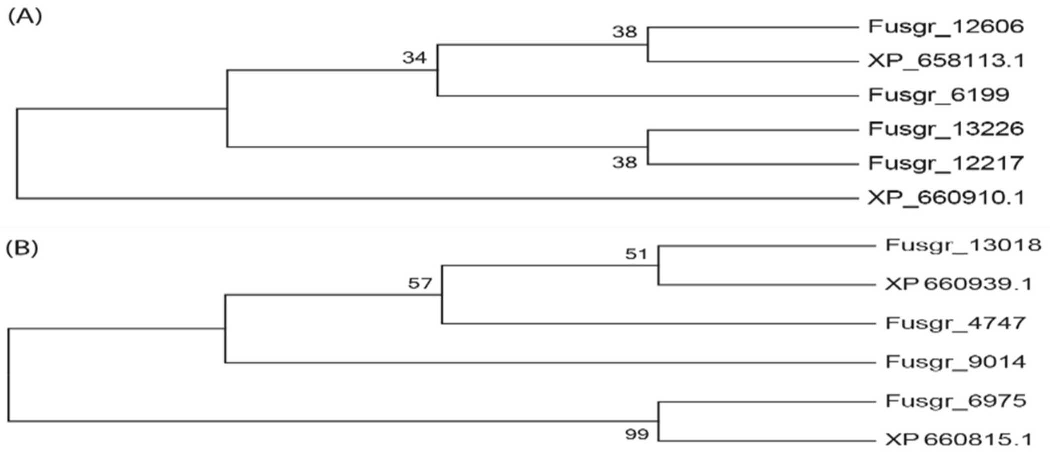

Figure S2. The phylogenetic relationship of (A) HCE gene family and (B) NPP1 gene family in *F. graminearum* genome.

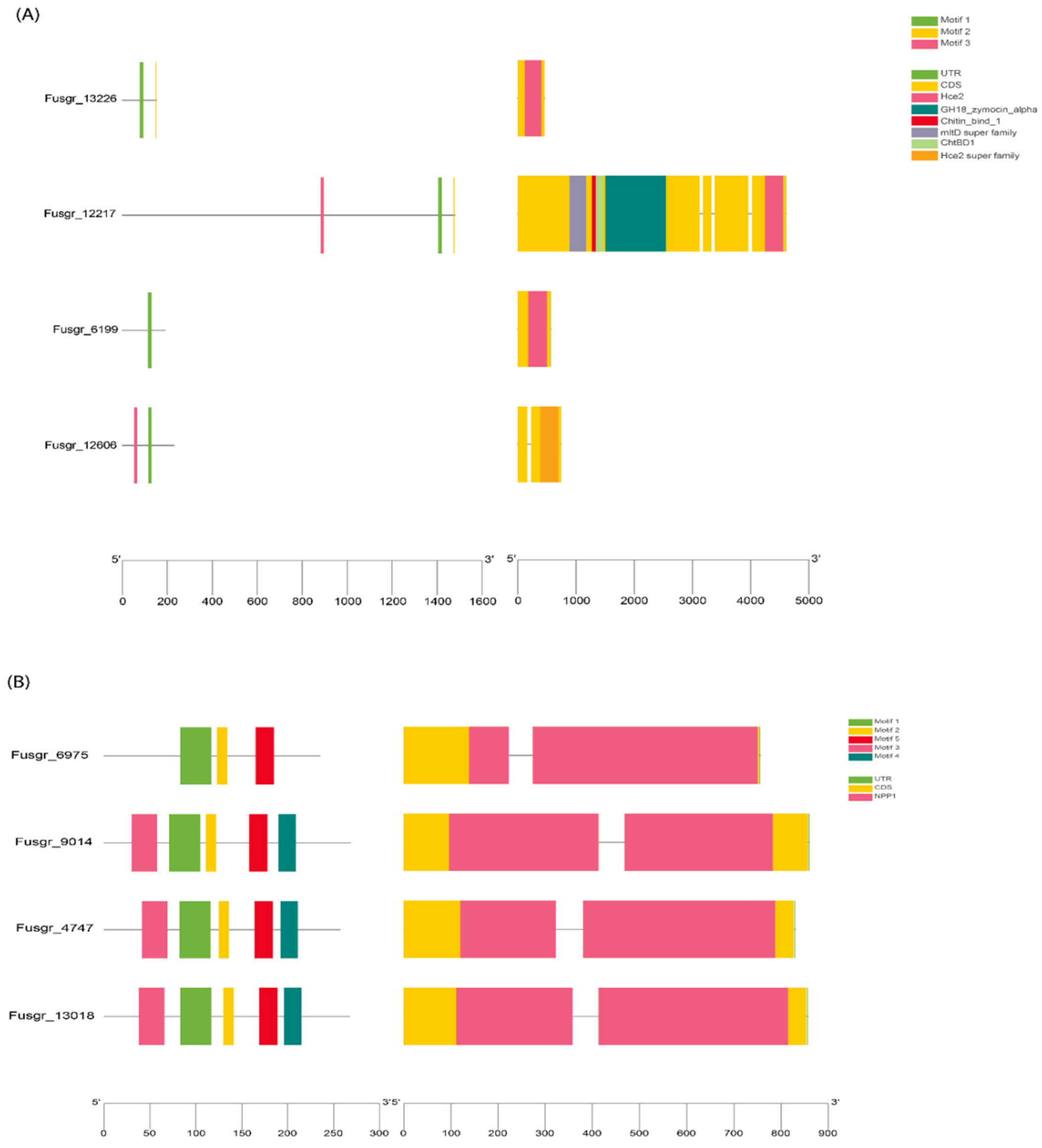

Figure S3. Structural and motif analysis of (A) HCE gene family and (B) NPP1 gene family in *F. graminearum*.

Table S1. The assigned m/z and peak intensities of detected homologues of LPs produced by *Bacillus* spp. FZB42 and TS1 at 15 °C and 25 °C.

Strains	Temperature	Mass (m/z) $[M+H]^+$	Compound	Peak Intensity
**TS1**	25 °C	1057.56	Bacillomycin	$5.49e^{6}$
		1071.58	Bacillomycin	$1.33e^{7}$
		1085.59	Bacillomycin	$5.90e^{5}$
		1008.65	Surfactin	$1.68e^{6}$
		1022.67	Surfactin	$1.80e^{5}$
		1435.76	Fengycin	$1.29e^{6}$
		1449.77	Fengycin	$3.07e^{6}$
		1463.78	Fengycin	$7.95e^{6}$
		1505.83	Fengycin	$1.23e^{6}$
	15 °C	1057.56	Bacillomycin	$3.78e^{6}$
		1071.58	Bacillomycin	$9.86e^{6}$
		1085.59	Bacillomycin	$2.93e^{4}$
		1008.65	Surfactin	$9.65e^{5}$
		1022.67	Surfactin	$8.29e^{3}$
		1435.76	Fengycin	$7.23e^{5}$
		1449.77	Fengycin	$1.74e^{6}$
		1463.78	Fengycin	$5.93e^{5}$
		1505.83	Fengycin	$8.32e^{5}$
**FZB42**	25 °C	1031.54	Bacillomycin	$2.28e^{6}$
		1045.55	Bacillomycin	$1.54e^{7}$
		1057.56	Bacillomycin	$9.00e^{5}$
		1059.57	Bacillomycin	$6.42e^{6}$
		1073.58	Bacillomycin	$4.14e^{6}$
		994.64	Surfactin	$6.31e^{6}$
		1008.66	Surfactin	$1.42e^{7}$
		1022.67	Surfactin	$5.60e^{6}$
		1036.67	Surfactin	$3.18e^{6}$
		1050.69	Surfactin	$2.94e^{6}$
		1435.76	Fengycin	$6.07e^{5}$
		1449.78	Fengycin	$2.49e^{6}$
		1463.78	Fengycin	$3.65e^{8}$
		1505.84	Fengycin	$1.21e^{6}$
	15 °C	1031.54	Bacillomycin	$9.24e^{3}$
		1045.55	Bacillomycin	$2.74e^{4}$
		1057.56	Bacillomycin	
		1059.57	Bacillomycin	$4.47e^{3}$
		1073.58	Bacillomycin	$8.69e^{2}$
		994.64	Surfactin	$3.71e^{4}$
		1008.66	Surfactin	$4.83e^{5}$
		1022.67	Surfactin	$5.21e^{4}$
		1036.67	Surfactin	$8.37e^{3}$
		1050.69	Surfactin	$1.71e^{4}$
		1435.76	Fengycin	$2.31e^{2}$
		1449.78	Fengycin	
		1463.78	Fengycin	$7.90e^{3}$
		1505.84	Fengycin	$6.53e^{2}$

Table S2: Primers used in this study for detection of genes and qPCR expression analysis.

**Detection of genes in *Bacillus* strain TS1**		
S.No	**Oligo Name**	**Sequence 5/ to 3/**
1	*eglS* (F)	ATGAAACGGTCAATCTCTATTTTTATTA
	eglS (R)	CTAATTTGGTTCTGTTCCCCAAATCAGT
2	*estA* (F)	ATGAAATTTGTAAAAAGAAGGATCATTG
	estA (R)	TTAATTCGTATTCTGGCCCCCG
3	*amyE* (F)	ATGTTTGCAAAACGATTCAAAACCTCTT
	amyE (R)	TCAATGGGGAAGAGAACCGCTTAA
4	*clpP* (F)	ATGAATTTAATACCTACAGTCATTGAACA
	clpP (R)	TTACTTTTTGTCTTCTGTGTGAGTCAAA
5	*sbp* (F)	GTGAACGCACAGTTATCGGCTAA
	sbp (R)	TCATACCCGTACACCTGCAGATTC
6	*Pvd* (F)	ATGAGACGGGAAGCGTTAAAGAATG
	Pvd (R)	TTAGTTATTCACATTTACGTCCTGTAACG
**Lipopeptide biosynthesis genes in *Bacillus* spp. FZB42 and TS1**		
1	*Bmy* (F)	CTGTCGGAGATGTCACAAGAA
	Bmy (R)	CGGCTTTCAGGCGTTTAATATC
2	*Fen* (F)	GTCGCAGAGCTTCAGAGAAA
	Fen (R)	GATGGACCGTCAGAAACAAGTA
3	*Sfb* (F)	GAACGCTATAAACGCGATGTG
	Sfb (R)	ATGATTGCTCCAGACGAGATAC
4	*rpsj* (F)	GAAACGGCAAAACGTTCTGG
	rpsj (R)	GTGTTGGGTTCACAATGTCG
***F. graminearum* pathogenicity-linked genes**		
1	*PAL* (F)	AGTCTATGGGCGGTAATGTTG
	PAL (R)	TTGTGCGAGTTGAGTAGCG
2	*SOD* (F)	ATTCCTCTGCCTAAATTCCCC
	SOD (R)	TCAGAAACTCGCTCAAACCAG

3	*CBH* (F)	GACGAGAACACCTACCAGATG
	CBH (R)	GGGCATCACAGTAACCAGTAC
4	*β-XSD* (F)	CGATGCTGGATTTGAGAATGATC
	β-XSD (R)	GTATTTGCCGTTCTTGTGGG
5	*TRI6* (F)	GCTACTCAGAATGCCCTCAG
	TRI6 (R)	TGATCTCGCATGTTATCCACC
6	*TRI10* (F)	TGGACCATGTTGACTATTCTCG
	TRI10 (R)	GTCTGTCGGTAGCCTTTGTAG
7	*PKS4* (F)	TGTGGGTTGGAAGTGTGAAG
	PKS4 (R)	AGGGTTTGGACTAGCGAAATG
8	*NPS1* (F)	CCATCGTCAACTGTGTCTACTG
	NPS1 (R)	CCAGTCTATCTCCAAAGCATCG
9	*NPS6* (F)	AAGCCAAGGATGAAGGAGATG
	NPS6 (R)	GACACGAGGTAGGGAATCTTG
10	*HCE1 (F)*	TTCCACCTTCGAGAACAAGTC
	HCE1 (R)	GCAGCGTTCATGTTTGATCC
11	*HCE2 (F)*	AAGATACACTCAGACGCCAAG
	HCE2 (R)	GCTCATATAAGACACCCATTCGG
12	*HCE3 (F)*	GATATCGTCAAGTCTGGTTCCTG
	HCE3 (R)	TTACACTTGACAGAACCACGAG
13	*HCE4 (F)*	CAGATGGCCTCTTACTTCCAG
	HCE4 (R)	AATTCACGAGCCCTATTCCG
14	*NPP1-1* (F	ACCTGAAGTACAAGCCTTTCG
	NPP1-1 (R)	GAGTACATGATGGCGTATCGG
15	*NPP1-2* (F)	CTGCCTTGAGTGGACATAGAC
	NPP1-2 (R)	CTTTGTGGTAGACTAGCTTGGG
16	*NPP1-3* (F)	ACAGCAACACCTATTCTCGC
	NPP1-3 (R)	TGAAGGAGCAACACGAAGG
17	*NPP1-4* (F)	CTATTGTAACTGTCCTCGCCG

	NPP1-4 (R)	TGTTACCGTCCTTGTCGATG
18	*Actin* (F)	TCTTCCAGCCTTCTGTCCTTG
	Actin (R)	AATGGAACCACCGATCCAGA
**Defense-related genes in wheat plant**		
1	*GNS* (F)	TTCCTACGAGTGTGCAATCC
	GNS (R)	GTTACCGATGTCGAGGATGAG
2	*HMGR* (F)	CGTTGTGGTTAGTTGGGTTG
	HMGR (R)	CAATCACGTCACCAACTTTCG
3	*PR-1* (F)	GGCTAATCTTTCCCAGGCG
	PR-1 (R)	TTGCAGTCGTTGATCCTCTG
4	*PAL* (F)	CTCACTTGGTCTGATCTCATCC
	PAL (R)	TGTTCATGCTCAGGGTCTTC
5	*SOD* (F)	TTGGGAATTGATGTCTGGGAG
	SOD (R)	ACAGCGGGAAACTCAAGAG
6	*LOX1* (F)	CCTTCATCCACACCATCACTAG
	LOX1 (R)	CTTGCTCTCGATCTCCACTAG
7	*MPK6* (F)	GACAAGATGGTACAGGGCAC
	MPK6 (R)	GTAGACGTAGCTGATGGACATG
8	*Actin* (F)	TGGATCGGTGGCTCTATTTTG
	Actin (R)	TTTGTAAGTCCCCTTCACCG
